# Providing appropriate health and social care for people with dementia or mild cognitive impairment in the criminal justice system of England and Wales: a thematic analysis of prisoner and staff interview data

**DOI:** 10.1186/s40352-024-00313-5

**Published:** 2025-01-20

**Authors:** Adam O’Neill, Leanne Heathcote, Laura Archer-Power, Stuart Ware, Jenny Shaw, Jane Senior, Katrina Forsyth

**Affiliations:** 1 University of Manchester, Manchester, United Kingdom; 2 Greater Manchester Police, Manchester, United Kingdom; 3 Restore Support Network, Exeter, United Kingdom

**Keywords:** Criminal justice, Dementia, Mild cognitive impairment, Prison, Older prisoners

## Abstract

**Background:**

The number of older adults entering the criminal justice system is growing. Approximately 8% of older prisoners in England and Wales have suspected dementia or mild cognitive impairment (MCI) and experience difficulties in everyday functioning, and disruption to their daily life. At present, no specific dementia/MCI care pathway has been implemented that is applicable and appropriate for use across different prisons in England and Wales. The aim of this paper is to explore the experiences of older adults with dementia/MCI in prison, and a range of key stakeholders, around the day-to-day issues faced by people with dementia/MCI and prison, healthcare, and third sector staff regarding the delivery of support for individuals with dementia/MCI.

**Methods:**

Thirty-two semi structured interviews were conducted with prison, local authority, and healthcare staff; peer supporters; third sector care providers; and individuals with dementia/MCI themselves, across five establishments, to provide multidimensional perspectives of dementia/MCI in criminal justice settings. The data obtained during interviews were thematically analysed.

**Results:**

From the data, six key themes emerged: (I) *ethical concerns* around trial, sentencing and detainment for people with dementia/MCI; (II) *An unforgiving prison* system, providing physical and social environments incompatible with supporting individuals with dementia/MCI; (III) *An unprepared workforce* requiring training in dementia/MCI. (IV) A *lack of collaboration* leading to sub-optimum management of the support needs of people with dementia/MCI in prison; (V) *Peer support ‘plugging the gap’*; and (VI) *staff ‘hands tied behind back’*.

**Conclusions:**

Results point towards a pressing need to develop more appropriate support systems for individuals with dementia/MCI throughout the criminal justice system. Ethical concerns around the judicial process for individuals with diminished cognitive capacity must be considered. Prison governors should examine ways to make the living environment more appropriate for these individuals, and a joined-up collaborative approach to health and social care should be adopted. Staff must be appropriately trained to support and identify individuals with dementia/MCI. Peer support schemes require formal evaluation, and training/oversight of these schemes should be comprehensive.

## Background

Approximately 982,000 people have a diagnosis of dementia in the UK, projected to rise to 1,400,000 people by 2040 (Alzheimer’s Society & Carnall Farrar, 2024). Estimates also suggest between one and two in every 10 people over 65 have mild cognitive impairment (MCI) (Alzheimer’s Research UK, 2021). Reported rates of progression of MCI to dementia range between 8% and 15% per year (Richardson et al., 2019), with some of the population with MCI at risk of developing dementia, and others possibly regaining some cognitive function (Ganguli et al., 2011; Matthews et al., 2008; Stephan et al., 2007).

In November 2023, there were 87,935 people in prison in England and Wales (Ministry of Justice, 2024). Data indicate over 17% of this population were older prisoners (Ministry of Justice, 2024), defined in this context as aged ≥ 50 years (Merkt et al., 2020). This group has increased substantially over the last two decades, as has their proportion within the prison population (House of Commons Justice Committee, 2020), reflecting worldwide trends (Psick et al., 2017). By mid-2024, prisons in England and Wales were nearing full capacity (Brader, 2024), which required the enactment of emergency measures, specifically early release for some prisoners, to alleviate the intolerable pressures placed on the justice system.

Recent research estimates approximately 8% of older prisoners in England and Wales have suspected dementia or MCI (Forsyth et al., 2020). People with dementia or MCI experience difficulties in everyday functioning and disruption to their daily life (Banerjee et al., 2006; Farias et al., 2006; Ray & Davidson, 2014). In prison, dementia and MCI is under-diagnosed (Brooke et al., 2020; Forsyth et al., 2020; Peacock et al., 2020). People with these conditions are often fearful of their environment and reluctant to ask for help. Access to specialised services is limited (Forsyth et al., 2020; Peacock et al., 2020; Treacy et al., 2019), meaning there are unique challenges to supporting people with dementia/MCI. There is also minimal specialist knowledge amongst staff, who often struggle to gain assessments from Memory Assessment Services (Brooke et al., 2020; Peacock et al., 2020).

Whilst older prisoners have substantial health and social care needs that often go unmet (Baidawi et al., 2016; Davies et al., 2023; Tucker et al., 2021), prison appears especially unsuitable for individuals with dementia (Forsyth et al., 2020; Moll, 2013). By design, prisons are rigid in their regime, restrictive of a person’s freedoms, and are more noisy than typical living environments (Forsyth et al., 2020); however, modifications to the physical and social environment, such as colour coded doors and greater flexibility in activity times are rare (Brooke et al., 2020; Dawes, 2009; Forsyth et al., 2020; Peacock et al., 2020; Stojkovic, 2007).

According to the principle of equivalence of care (Till et al., 2014), prisoners should receive comparable levels of health and social care provision to the general population. All National Health Service (NHS) and Department for Health and Social Care (DHSC) standards therefore apply to prisons, including the principles comprising the Care Act 2014 (UK Legislation, 2014). Nonetheless, the standards outlined in this legislation remain largely unmet in prisons across England and Wales (Forsyth et al., 2020; Tucker et al., 2018), and a national strategy for an ageing prison population is long overdue (O’Neill & Falvey, 2023), despite being recommended by parliament in 2020 (House of Commons Justice Committee, 2020).

Presently, no specific dementia/MCI care pathway applicable and appropriate for use across different prisons in England and Wales has been implemented (Forsyth et al., 2020). Research suggests dementia friendly community principles are acceptable in prisons (Treacy et al., 2019); however, the implementation of new approaches to mental health in prisons can be challenging (Caulfield & Twort, 2012), with equivalence of care between prison and community mental health care considered particularly difficult to achieve without highly targeted funding and provision. Therefore, research must endeavour to develop pragmatic, cost-effective, and focused solutions to support staff and prisoners. Overall, current understanding and awareness of dementia and MCI among prison staff is low (Purewal, 2020), and there is minimal research on training needs, preferences, and content. There is also a need to examine and clarify the role and training of peer supporters in prison, who typically perform non-intimate care tasks such as cleaning cells and helping individuals mobilise (Walton et al., 2023).

The aim of this paper is to explore, via semi structured interview, the experiences of older adults with dementia/MCI in prison, and a range of key stakeholders, around the day-to-day issues faced by prison, healthcare, and third sector staff regarding the delivery of support for individuals with dementia/MCI. This speaks to a current gap in evidence needed for the development of specialist care pathways and training packages for dementia and MCI in prisons.

## Methods

### Sites

Five sites were purposively selected to represent different category prisons across the male estate in northern and midlands England. They were: an adult-male category B prison; an adult-male category B local prison; an adult-male category A prison; an adult-male category C prison for individuals convicted of sexual offences; and an adult-male category C prison with specialist vulnerable prisoner capacity, including a dedicated wing for adults with higher health and social care support needs. Peer support schemes where in place at each of these establishments, though models of delivery varied.

### Sampling

Healthcare and operational staff at research sites were responsible for identifying imprisoned participants based on the following inclusion criteria: (I) be resident in one of the selected sites; (II) have suspected dementia or MCI; and (III) be able to converse in English. The clinical, behavioural, and social presentation of these individuals was then discussed amongst researchers, healthcare, and operational staff. Through these discussions, purposive selections were made such that the final sample was representative of differing symptomatic severity and support requirements. Those selected where then approached to participate.

When possible, individuals with suspected dementia/MCI were also asked to identify individuals (peers or staff) who they felt could provide valuable insight into how they managed their needs on a day-to-day basis, such as those involved with their healthcare or assisting with tasks of daily living. This helped achieve a *360-degree* insight into specific prisoners. Research team contacts and staff at sites were also used to identify additional participants in supporting roles and managerial staff, purposively selected to represent a wide range of roles involved with the support of people with dementia/MCI.

In total, 32 semi structured interviews were conducted with prison, local authority, and healthcare staff; peer supporters; third sector care providers; and individuals with dementia/MCI themselves, to provide multidimensional perspectives of dementia/MCI in criminal justice system (CJS) settings. Of the 32 interviews conducted, nine were with adult males in prison with suspected dementia/MCI (at least one at each of the participating sites), three were with peer supporters in prison, and two were with people who shared a cell or wing with a person with suspected dementia/MCI; the remaining 18 interviews were with staff acting in a professional capacity to support people with dementia/MCI in prison. Specific roles included: prison officer (various grades/sub-roles), governor, deputy governor, general practitioner, psychiatrist, healthcare manager, social worker, mental health worker, and probation officer.

### Consent

For those individuals with suspected dementia/MCI, informed consent was sought using Dewing’s (2007) widely accepted five-step Process Consent method. First, researchers ensured they approached consent knowing some relevant biographical detail of the individual to be consented and that the relevant prison authorities and wing staff had given permission for the person to be approached. Second, researchers trained in the Mental Capacity Act (MCA) (UK Legislation, 2005) established the basis of consent, assessing capacity using the two-stage process outlined in the MCA: (I) Do they have the capacity to make the decision – are they understanding, retaining, able to weigh up and communicate their decision? (II) Is their inability to make the decision the result of functional impairment in the mind or brain? If researchers assessed the individual possessed capacity, they would then move on to step three of the process consent method, initial consent. In this step, participants were presented with and had explained to them a participant information sheet (PIS), as well as being given the opportunity to ask any questions. Written consent was sought from participants that they fully understood the details of this and were happy to participate in the study. Fourth, consent was monitored as an ongoing process. This entailed ensuring the individual did not lose capacity throughout the interview, or that the participant would want to withdraw consent. Lastly, feedback was provided to relevant support networks such as wing staff or healthcare professionals that the interview had taken place and, if needed, any concerns would be relayed, in line with agreed confidentiality protocols.

In the event it was felt an individual was incapable of giving informed consent due to having diminished capacity, an attempt would be made to identify a “Personal Consultee”, as defined by the MCA, to advise on the individual’s participation. This might be a relative of the participant, whom they nominated, or an appropriate independent consultee, typically a clinician or healthcare worker from within the prison. For this study, no approached participants lacked the capacity to consent, therefore this fallback process was not utilised.

Staff participants were similarly consented having read a PIS and been given the opportunity to ask any questions. Written consent via consent form was then sought before interviews could take place.

### Semi structured interviews

Individuals in prison were asked about their experience of dementia and the support they have received throughout their contact with the CJS. Questions centred on five topics: Identification, e.g., ‘how were your memory/thinking problems first picked up?’; arrest and court, e.g., ‘how did your memory/thinking problems affect in court/whilst at the police station?’; prison entry, e.g., ‘can you describe how your memory/thinking problems affected you when you first came into prison?’; prison life, e.g., ‘have any day-to-day activities been particularly difficult?’, ‘what support has been provided?’; and if applicable, release, e.g., ‘how do you feel plans for your release are going?’.

Staff members were encouraged to reflect on their responsibilities for this prisoner group in the context of the wider environmental and organisational setting in which they work. This entailed consideration of their training needs, role, personal confidence, and competencies. Questions included, ‘what helps you or would help you to identify and support these individuals?’, ‘what further training/support do you feel you/your staff need (if any)?’, ‘what kind of content do you think should be included in the training?’, and ‘what challenges do you face in identifying and supporting these individuals?’.

The interviews all took place in person, within prisons. Participants were only interviewed once, even if interviews were shortened for operational reasons (staff) or loss of capacity/distress/confusion (people with suspected dementia/MCI). They were audio-recorded and lasted between 20 min and an hour. All recordings were professionally transcribed before analysis.

### Qualitative analysis

The data obtained during interviews were thematically analysed following Braun and Clarke’s (2006, 2012) six-step method of thematic analysis:

A. Familiarisation with the data: The analysis began with readings and engagements with the transcripts to gain an understanding of the content and context.
B. Generating initial codes: Following familiarisation, initial codes were systematically generated to capture meaningful segments of the data. Each code represented a distinct concept or idea.
C. Generating themes: Codes were then organised to identify potential themes. Themes emerged as patterns or clusters of codes that shared commonalities or conveyed significant aspects in the data.
D. Reviewing themes: Identified themes were reviewed and refined to ensure accurate representation. This involved scrutinising each theme to assess its coherence, relevance, and distinctiveness.
E. Defining and naming themes: Following review, themes were defined and named to encapsulate their content and meaning.
F. Writing up the analysis: The final step involved synthesising the thematic findings into a coherent narrative. The analysis was presented in a structured format, where each theme was split into coherent sub-themes and re-enforced with researcher exposition and participant quotations.

Steps D-F were completed collaboratively by researchers in person or via online video conferencing, including directly with a senior qualitative researcher.

## Results

**Fig. 1:**
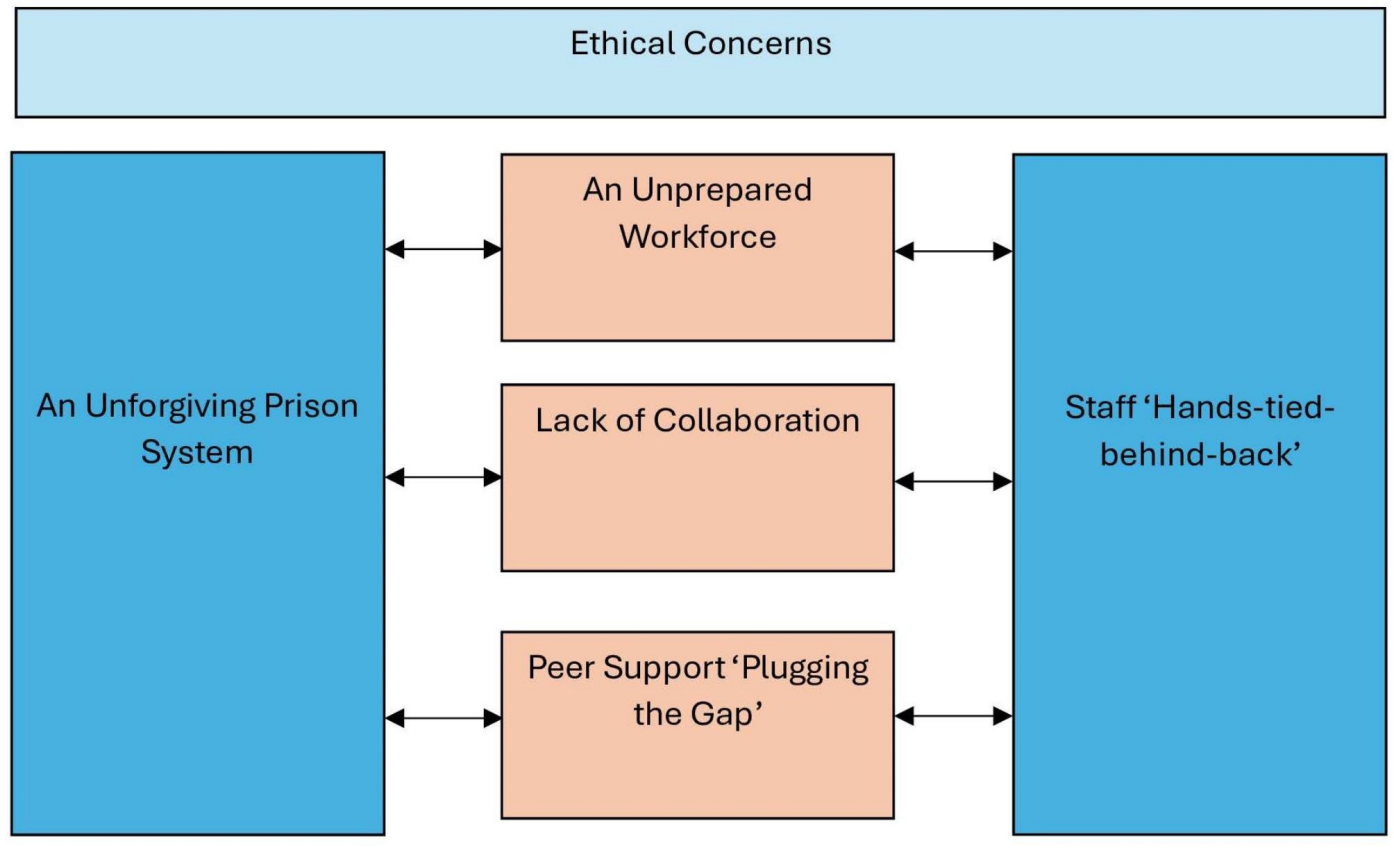
Inter-connected themes

Six inter-connected key themes emerged from the data (Fig. 1). These were: (I) ethical concerns; (II) an unforgiving prison system; (III) an unprepared workforce; (IV) lack of collaboration; (V) peer support ‘plugging the gap’; and (VI) staff ‘hands-tied-behind-back’.

### (I) Ethical concerns

Ethical concerns were prominent across the data set. Broadly, they fell into two sub themes: sentencing, and the morality of detainment given dementia.

#### Sentencing

Concerns around sentencing were repeatedly raised by those with dementia/MCI. Several felt that their sentence was unjust or raised concerns regarding the fairness of the legal proceedings they had been part of. One individual with dementia described how, despite two psychiatrists assessing that they would be unable to understand the trial, the courts proceeded nonetheless, bringing into question fitness to plead and stand trial:

*I saw two psychiatrists; one for prosecution*,* one for the defence; and they both put in their report they didn’t think I would be able to understand the trial. That didn’t make any difference to the judge. He turned round and said*,* as far as I’m concerned*,* this case needs to go up to trial*,* which it did*.

(Person with dementia/MCI A)

A further ethical concern appeared regarding sentence length. Multiple references were made to sentences which potentially imprisoned individuals for life without being categorised as life sentences. Concerns around this were expressed by staff and individuals with dementia/MCI alike. One explained that their sentence, given their circumstances, covered a significantly longer time period than they would expect to live. The judge had issued them a de-facto life sentence, without their sentence or crime being categorised as such:

*You think I’d serve 20 years at my age? No chance at all*,* I could never serve 20 years*,* that will make me 108*,* won’t it? I’ll never reach 108*.

(Person with dementia/MCI B)

#### The morality of detainment given dementia

The morality of detaining individuals with more advanced dementia was repeatedly called into question by participants. In particular, concerns were raised over individuals who, due to dementia, lacked awareness of their surroundings and temporality. For staff and imprisoned individuals alike, their condition raised complex ethical concerns as to whether an individual whose cognition is deteriorated to the extent that they lack awareness of self, time, or surroundings, should in fact be treated by the CJS as if their cognition were still functioning as when the crime was committed and remain in prison. For example, some individuals were unclear on the reason for their incarceration, how long they had been in prison, and one individual appeared unable to consistently keep track of the present day:

*I get up every day and I don’t know what day it is. I know what day it is today*,* it’s Thursday*,* but I didn’t know when I woke up and I didn’t know an hour after I’ve woke* *up*.

(Person with dementia/MCI A)

### (II) An unforgiving prison system

References to the unsuitability of the prison system were widespread throughout the data. Five sub-themes emerged which all spoke to how unforgiving the prison system was for those with dementia/MCI. These were: prison system rigidity; physical prison environment; complex management of symptoms; locked up and confused; and alternatives to custody.

#### Prison system rigidity

The lack of flexibility in the regime was thought to prevent individuals with dementia/MCI living a comparative life to that of their peers in prison and in the community. Individuals with dementia/MCI and their supporters spoke of difficulties completing time-bound tasks, where no additional time allowance is granted for individuals with dementia/MCI compared to their imprisoned peers, despite their condition potentially slowing their mental and physical functioning. This places extra burden on individuals with dementia/MCI who may not be able to complete tasks as quickly. One individual described how their ability to wash was impeded by a strict morning regime which required them to complete multiple tasks in a short time frame:

*Some mornings you can’t* *[shower]…* *they get you out of the cell about twenty to eight*,* and ‘til twenty past eight you’ve got to shower*,* exercise*,* meds*,* toast*,* it’s all got to be done in that time*.

(Person with dementia/MCI C)

Also notable was the inability to adapt the regime to the needs of individuals when governor orders were issued. Across staff there was an evident sense of frustration that this prevented them from supporting individuals with dementia/MCI:

*Governor orders they’ve got to be banged up*,* that’s it*,* bang up.*

(Prison Social Worker A)

*If there was… a bit more leeway with things*,* we’d be able to help them a bit more*.

(Prison Officer A)

An emphasis on security also appeared to prevent individuals from attending external healthcare appointments and limited response capacity in emergencies. Barriers to accessing emergency or specialised care based on establishment staffing are not present for individuals in the community, meaning those in prison are receiving care which is held to a different or lesser standard than in the community. This was raised as problematic by multiple professionals, with one explaining:

*If there’s any incidents*,* we can’t send anybody out (of the prison). If they’ve got low staffing that day*,* you can’t send anybody out*.

(Mental Health Worker)

#### Physical prison environment

The unsuitability of the physical prison environment was also highlighted. Individuals with dementia/MCI and their supporters spoke of issues navigating the prison environment safely. They also described few adaptations to the environment and that prisons were missing necessary equipment to support individuals with higher, more complex needs. This appeared to present individuals with dementia/MCI with challenges of daily living that are not faced by their peers in prison and would not be faced were the individuals with dementia/MCI living in the community, where a person’s capacity to access or arrange accommodations is less restricted:

*To walk in them white lines in a wheelchair it’s like needing a 4 × 4*,* because the ground’s all uneven. There’s potholes everywhere*.

(Peer Supporter A)

*I go for showers. I said to them (…) I want to have a seat because I’m frightened of falling*,* because I’m at that stage now that I’ve got to cling on to things when I walk.*

(Person with dementia/MCI B)

*I can’t turn myself on the steps*,* I’ve got to keep going up and then…if I’ve got halfway up the steps and think I don’t need to be up here*,* I can’t turn round on the stairs because if I do*,* I’ll go over.*

(Person with dementia/MCI E)

*He was jerking excessively to a point where he nearly ended up on the floor quite a few times… He was completely different to what we’ve seen in the past couple of days with him. [The local authority’s] response was that he [would] be in residential care if he weren’t in prison. There was no suitable equipment*,* there was no support in the cell to meet his needs*.

(Social Worker B)

#### Complex management of symptoms

Common secondary symptoms associated with more severe dementia/MCI appeared particularly difficult to support. This was expressed by participants in managerial positions, those providing direct practical support, and individuals with dementia/MCI. One Deputy Governor highlighted how symptoms impacted on the individual with dementia/MCI’s day-to-day life, as well as their peers:

*We had a situation where his behaviour was impacting on his cell mate*,* so we had to move him out. [He had] a tendency to soil himself*,* and when he’s in communal areas we’ve had to lock down the whole wing to clean; that impacts on the other people’s time out of their cell*.

(Deputy Governor)

Individuals with dementia/MCI described how their symptoms, and the effect of their symptoms on others, negatively impacted their psychology, with many symptoms leading to both *in the moment* and extended distress or anxiety. In particular, individuals with dementia/MCI reported increased anxiety and prolonged distress around how others might react to their presentation, given that prison is an environment where harassment and bullying is common. One individual described how this impacted their thoughts and behaviours; and a support worker empathised with their worry:

*You think*,* oh God*,* why are you all picking on me? What have I done? It makes you feel a bit unnerved. You just go into your own little shell a bit then. It’s terrible*.

(Person with dementia/MCI D)

*It’s quite a scary world for somebody with dementia to come into prison*.

(Social Worker C)

#### Locked up and confused

A recurring concern raised by individuals with dementia/MCI and staff was the amount of time individuals were locked in their cell. Particularly worrying was that, due to the forgetfulness experienced by individuals with dementia, the individual might be unaware of why they were being locked up, leading to significant distress:

*It’s very hard to explain to somebody with dementia who doesn’t even know why they’re here [or] what they’ve done. Depending on the level of dementia (…) you’re having to explain to them*,* well you’ve got to stay in your cell now for a certain amount of time*,* if you need help press your bell. But then they’ll be pressing their bell every five minutes ‘cause they’ll have forgotten*.

(Prison Officer B)

#### Alternatives to custody

Comments to the effect that individuals with more severe dementia were not receiving support equivalent to that which they would receive in the community were common. Operational limitations imposed by the prison, including the regime and staffing, were thought to make this impossible, and several participants, including at the managerial level, suggested that specialist accommodation was required that might facilitate support closer in delivery to that provided in the community:

*They (people with more advanced dementia) would probably be looked after in a nursing home*,* 24-hour care… in a prison it’s more difficult*,* because we’ve got reduced operational staff on duty at nigh*t. (…) *further down the line we will see some specialist*,* discreet accommodation*.

(Governor)

The idea of specialised units was suggested by multiple contributors. Some thought this facility should be outside of prison walls, however, more commonly participants described a dedicated wing, or part of the prison, with specialist capacity to address complex health and social care needs. One officer described a wing dedicated to prisoners who required more support:

*In an ideal world*,* [the wing would] be sectioned off with all disability cells and maybe extra staff*,* maybe even a medical member of staff down there to assist*.

(Offender Supervisor)

### (III) An unprepared workforce

Participants highlighted that the prison workforce is not adequately trained in or aware of the needs of individuals with dementia/MCI. Commentary on this lack of preparedness fell into three sub themes: training development needed; interpersonal skill; and more dementia awareness needed.

#### Training development needed

There was an almost universal acceptance that staff in prisons do not receive the required support or preparation to work with individuals with dementia/MCI. Operational staff acknowledged their limited capacities in identifying the signs and symptoms of dementia/MCI, which they felt limited their response to individuals with dementia/MCI:

*There’s a lot of dementia*,* I feel like*,* but we don’t have that knowledge or training to be able to deal with it efficiently*.

(Prison Officer C)

*[The biggest challenge is] not having the actual proper training for it (dementia); having to deal with it as it comes along*,* not really knowing what to expect*.

(Prison officer D)

Officers’ initial training was considered overall inadequate, with many operational staff deriding the extent to which it prepared officers for the reality of their role. Dementia/MCI was noted by one senior officer as a key absent aspect of new officer training:

*Officer training in general is far too short. Effectively eight weeks training to deal with the mental health issues*,* the behavioural issues*,* the security… This sort of thing (dementia/MCI) should be discussed in the training*,* and it’s just not*.

(Safer Custody Officer)

#### Interpersonal skill

Interpersonal skill describes the skill required to effectively communicate, interact, and support individuals and groups. It was noticeable in the data that health and social care staff perceived prison officers’ approach to individuals with dementia/MCI as lacking in interpersonal skill. This appeared tied to the idea that officers might assume that non-adherence to prison rules is wilful behaviour or non-conformism, rather than a side effect of the confusion, disinhibition, and disorientation individuals with dementia/MCI commonly experience:

*Officers tend to automatically assume that if somebody isn’t conforming or acting in the normal way*,* that it is a disciplinary issue*.

(Head of Healthcare)

Nonetheless, officer responses pointed towards the need to view their interpersonal approach in a nuanced way. Training in interpersonal skill might well be insufficient, such that some officers enter the role unprepared to engage in meaningful and supportive interaction with individuals with dementia/MCI. However, it may also be that due to officers’ lack of training in identifying signs of dementia/MCI, they are unable to deduce whether their interpersonal approach is appropriate. One officer suggested that if they were more informed on the development of dementia/MCI, officers may not resort to a disciplinary approach by default.

*If I knew what to look for whilst they’re going through*,* or getting worse in their illness*,* then it would be so much easier for us; because sometimes… you can have someone who’s really*,* really quiet and they just go up the wall and whatever else; if we didn’t know what to look for – and some of us don’t – we’re just going to think*,* oh*,* they’re misbehaving*,* we’re going to have to deal with them like this*.

(Prison Officer D)

Participants in operational roles felt that efforts to develop interpersonal skill in officers therefore should not just focus on how to interact with individuals with dementia/MCI. It was perceived that they should also aim to equip officers with the knowledge to identify behaviours which may be caused by dementia/MCI and appropriate approaches for when an individual who might have dementia/MCI exhibits those behaviours.

#### More dementia awareness needed

Developing the approach of officers was believed to require not just improving the training for new officers, but also raising awareness of the support needs of people with dementia/MCI amongst current officers and other wing staff. One contributor stressed that everyone on a wing should be aware of dementia and how it can affect people:

*It’s not just to do with officers*,* it’s to do with everybody else that’s on the wing*,* we all should be made aware that certain people have problems and might need help.*

(Prison Officer E)

### (IV) Lack of collaboration

It was clear from the data that a lack of collaboration inhibited the support provided to people with dementia/MCI in prison. This emerged as three sub-themes: no clear pathway; strained relations; and siloed working.

#### No clear pathway

It was evident across the data that diagnosing and providing the necessary care for individuals with dementia requires collaboration within prison teams, as well as with mental health providers and community services. However, descriptions of the process for identifying and supporting individuals with dementia/MCI appeared complex, convoluted, at times speculative, and lacking a defined pathway:

*[People with dementia/MCI] usually get identified in reception… When they come on the wing*,* we do identify if they have any signs. We’ll speak to them and see if they recognise it themselves. If not*,* then we can refer them to mental health*,* or just our nurses in general. They’ll come and see them*,* or they’ll go over to the doctor*,* who can assess them. And then the doctor will either inform us they may have*,* they may not*,* and then we can work with them on the wing.*

(Prison Officer A)

#### Strained relations

There also appeared to be strained relations between prison staff and other teams tasked with providing support for those with dementia:

*We have to have a good working relationship with all the different people and teams*,* it’s all our responsibility; and it frustrates the hell out of us when certain teams ain’t doing certain things*.

(Key Worker)

It was acknowledged that the relationship between prison staff and mental health teams operating throughout the prison estate, who might provide therapeutic and psychological support for psychiatric disorders, substance misuse, and mental distress, was not developed enough for them to operate effectively:

*We need liaisons from our mental health teams*,* for them to actually say to staff… you’ve got this prisoner*,* he’s got this problem*,* this is how you deal with it. And I don’t think we’ve got that sort of relationship*.

(Safer Custody Officer)

#### Siloed working

Implicit references to siloed working were notable. A lack of connectivity between different prison-based services and communication issues were highlighted. Siloed working appeared to represent a highly limited approach to dementia care, where a multidisciplinary biopsychosocial approach is required in diagnosis, monitoring, and support. Multidisciplinary dementia care teams would optimally require psychiatric, pharmacological, social care, and psychological input. Contextual information, imaging, and screening all come together to present a picture of a person’s dementia; however, if these aspects remain in isolation, the picture remains incomplete, leading to sub-optimally planned support. Concerns to this effect were echoed by various professionals:

*[We need an] MDT (multidisciplinary teams) approach to take care of the individuals… but it just feels that it could be a bit more joined up.*

(Head of Healthcare)

*[To stop people’s symptoms being missed] they need to have better communication with each service*.

(Social Worker A)

### (V) Peer support ‘plugging the gap’

Peer support was a recurring feature within the data set. Discussion of peer support was separable into four sub-themes: staff endorsement; prisoner endorsement; lack of peer role clarity; and lack of recruitment and training clarity.

#### Staff endorsement

Peer support or ‘buddy’ schemes were seemingly widespread and aiming to deal with a variety of health-related and behavioural challenges, including substance misuse and healthcare. Staff appeared to endorse such schemes being used to support individuals with dementia, and gave examples of peer supporter activities:

*We have healthcare reps… we’ve got recovery champions that deal with substance misuse… So*,* there’s no reason why we can’t have dementia friends’ prisoners as well*.

(Head of Healthcare)

*They just look after their daily needs*,* like bring their food to them*,* change their bed*,* make sure they go for a shower*,* just speak to them… just like a carer on the outside but in the prison. So*,* I think that’s very helpful*.

(Prison Officer C)

#### Prisoner endorsement

Individuals with dementia receiving peer support described this positively, highlighting the value of the practical assistance they received:

*They come in and do things I’m struggling with like mopping the cell out for me… and doing certain jobs for me*,* doing my bed*,* which I have difficulty with. And pushing me over to meds when I need to go in my wheelchair*.

(Person with dementia/MCI D)

#### Lack of peer role clarity

Nonetheless, there appeared a notable lack of clarity as to what the role of peer supporter should entail, leading to evident blurred boundaries for the supporters and individuals receiving the support. Staff in particular spoke of supporters going beyond what is expected of their role:

*The carers*,* bless them*,* they have to go that little bit extra and do a little bit more sometimes.*

(Safer Custody Officer).

*I just asked one of the carers on the wing – even though he wasn’t getting paid for it – if he would just keep an eye on him and show him how to use the phone every other day*.

(Offender Supervisor)

#### Lack of recruitment and training clarity

A lack of peer role clarity may follow from a lack of rigour and clear role demarcation in the training of peer supporters, combined with limited knowledge of the training on the part of staff:

(Social Worker B)

*I think [peer supporters] could be doing with more training; but again*,* it’s all down to training.*

(Safer Custody Officer)

However, generally there was notable uncertainty as to the training’s content, with attempts to describe the training by staff unclear or confused:

*Carers do certain training*,* and the training they do basically: wheelchairs; they may go in and help them*,* I wouldn’t say actually dress them*,* they don’t actually dress them*,* but they’ll help them; they’ll remind them about going and getting their meds and things like that*.

(Safer Custody Officer)

One peer supporter, working on a healthcare wing, even suggested they had received no training, which left them feeling unprepared to perform their role:

*I’m not [prepared]…. we haven’t had training whatsoever, it’s only through my life outside of the job that I understand what some of these (people) are going through.*

(Peer Supporter B)

Additionally, descriptions of the recruitment process appeared simplistic given the nature of the role, and anecdotal success rate of applicants was noticeably high:

*They volunteer themselves to become a carer*,* and once they become a carer it goes through Safer Custody to see whether they can do the job*,* or whether they’ve got the right ability; and it depends on what (risk assessment) colour they are*,* whether they’re amber or green. If they’re green*,* nine times out of ten they will become [a peer supporter]*.

(Safer Custody Officer)

### (VI) Staff ‘hands-tied-behind-back’

Participants described that health and social care and operational staff were operating with their hands tied behind their back when supporting people with dementia/MCI in prison. There were two sub-themes around this: identification and diagnosis and lack of specialised care.

#### Identification and diagnosis

Identification and diagnosis appeared to rely on patchy, ill-defined processes. Often, prisons seemed to rely on cognitive impairment being picked up in reception and later via self-report or officer observation. However, as found in themes III and IV, officers do not have the necessary training, collaborative relationships, or understanding of dementia/MCI to fulfil this role:

*Unless somebody has previously been diagnosed or is self-aware to say*,* I have an issue – I don’t know how long the screening is in reception*,* but it’s very minimal – they’re unlikely to pick up any signs during the process*.

(Governor)

Relying on reception screening and officer observation to identify dementia/MCI also does not allow consideration of the key contextual factors involved in reaching a diagnosis, which may be obscured by the prison system’s rigid regime and capacity to support daily functioning. For example, prisons ensure people eat three meals per day, making it more difficult to detect if an individual is forgetting to eat:

*In memory assessment you really depend on the collateral information in terms of how they are functioning*,* how this memory problem is interfering with day-to-day life*. (…) *[prison is] an artificial situation… So*,* they’re not really displaying these key skills which would give you an idea of how this patient’s affected by their memory problem*.

(Geriatric Psychiatrist)

#### Lack of specialised care

Attempts by health and social care providers to support populations with dementia in prison were often considered sub-optimal. Specialised medical care for dementia/MCI, such as admiral nursing, specialist geriatric psychiatry, access to diagnostic equipment, or a dedicated role for the management of dementia or MCI patients, was reported as absent across most establishments:

*We don’t have access to CT scanners… so everything has to go out (be referred to community services) within the restraints of the prison system*.

(Mental Health Worker)

*We don’t currently have a role specifically looking at patients with cognitive impairment*.

(Head of Healthcare)

*[We could make services better by] having a kind of geriatric doctor in*,* someone that was really experienced in it and knew exactly kind of what we should be doing*.

(Mental Health Worker)

## Discussion

### Summary of findings

The six key themes point towards a pressing need to develop appropriate support systems for individuals with dementia/MCI throughout the CJS. Ethical concerns arose around trial and sentencing for people with dementia/MCI, specifically: whether the justice system handles questions around fitness to plead appropriately; the issuing of long, beyond expected end of life sentences for older adults; and the detainment of individuals with diminished cognitive capacities. In addition, the rigidity and unsuitable physical environments found within the prison system were thought to be incompatible with supporting individuals with dementia/MCI. Time-bound tasks were reported as challenging for people with dementia/MCI, and the effect the symptoms of dementia/MCI had on other prisoners caused the person experiencing the symptoms significant anxiety around bullying and victimisation. Likewise, locking people with dementia/MCI behind cell doors for extended periods appeared to be a confusing and distressing experience for these people – a concern similarly raised by staff. Managerial staff also spoke of the challenges accommodating people with dementia/MCI on regular wings. Alternative specialist accommodation was considered as a potential solution.

Further training for the prison workforce in dementia/MCI was a priority for staff interviewed. Specifically, operational prison staff acknowledged the need for more comprehensive training in identifying potential signs and symptoms of dementia/MCI and in interpersonal interaction with this group. More widely, awareness of dementia/MCI throughout the CJS needs to be raised. A lack of collaboration was thought to contribute to sub-optimum management of the support needs of people with dementia/MCI in prison. Strained relations between teams and a lack of a defined care pathway for individuals with dementia/MCI has led to siloed ways of working which are entirely at odds with the multidisciplinary approach needed to diagnose and support people with dementia/MCI. Peer support schemes currently buttress this lacking health and social care support; they were appraised positively by those receiving support and staff alike, however, recruitment, training, and monitoring processes appear to require significant development. Lastly, a lack of access to specialised care and patchy, non-formalised identification processes means health and social care providers are attempting to diagnose and subsequently meet the support needs of those with dementia/MCI with their hands effectively tied behind their back. Limited access to diagnostic services and technology, a lack of specialist staff, non-standardised identification practices, and the challenges of diagnosis in the prison environment, all present unique obstacles to supporting dementia/MCI not found in the community.

### Comparisons to literature

#### Ethical concerns

Given that dementia/MCI can decrease cognitive functioning, recollection, and situational awareness (Farias et al., 2006; Ray & Davidson, 2014), individuals with more severe dementia/MCI may lack the capacity to meaningfully engage with legal processes. Court proceedings for individuals with dementia/MCI that do not make allowance for potential diminished cognitive capacity may not meet the standards of a fair trial (Equality and Human Rights Commission, 2021), due to the individual’s potential inability to “understand in detail the nature and cause of accusations”, and “defend themselves”. At present, it is the responsibility of the courts to decide the extent to which dementia/MCI is a prominent consideration during trial and sentencing (Rethink Mental Illness, 2023; Sentencing Council, 2020). However, current guidance which allows judges to weigh the value of psychiatric reports may not go far enough to guarantee a fair, ethical judicial process for these individuals. Only a very small number of defendants in England and Wales are found unfit to plead (Brown et al., 2018), with 0.1% of Crown Court defendants being found unfit to plead between 2002 and 2014 (Mackay, 2016). Changes have been proposed by the Law Commission’s (2016) *Unfitness to Plead* report, which made several recommendations, including: modernising the test for unfitness to plead and align it with current psychiatric practice and understanding; extending the unfitness to plead procedure to magistrates’ and youth courts; training for judges and solicitors in identifying where support is needed; and a statutory entitlement to assistance for a defendant when required. In 2023, the UK government published their response accepting most specific recommendations (UK Government, 2023). It is important that these recommendations are enacted quickly and comprehensively.

Concerns also arise as to how appropriate it is for courts to issue *disguised* life sentences, where the sentence length issued to an older individual with dementia/MCI is beyond the timeframe through which that individual might reasonably expect to live. Sections 274 and 285 of the Sentencing Act 2020 (UK Legislation, 2020a, 2020b) stipulate the conditions for life sentences. However, there is a danger that these conditions might be bypassed in the issuing of sentences that imprison a person beyond their life expectancy without being life sentences. Nonetheless, sentence length must be balanced by courts against victim and public expectation of appropriate punishment for crimes considered by society and recognised in law as more severe, including sexual offences. The House of Commons Justice Committee (2020, pp.8–9) report on the *Ageing Prison Population* acknowledges that in recent years, a rise in convictions for historical sexual offences means increasingly older prisoners are “prisoners sentenced for the first time later in life for a long sentence”. The report indicates that: “45% of men imprisoned aged 50 or over are serving sentences for sexual offences, including historic offences; for those aged over 70, the figure is around 80%” (House of Commons Justice Committee, 2020, p.9). The high prevalence of older individuals with this offence profile, and dementia incidence increasing with age (Matthews & Brayne, 2005), likely accounts for the presence of this concern within the present data set; yet the significant moral and ethical challenges inherent in this situation cannot be ignored.

By the principles of retributive justice (Walen, 2023) society might demand accountability and sentencing proportionate to the crime, irrespective of the perpetrator’s age. However, this notion of justice is subject to wide-ranging criticism (Mayer, 2014), including concerns around the value or lack thereof in being motivated by retribution, the moral right to exercise retribution, and the limitations of this approach to address the underlying issues that lead a person to crime. In addition, incarceration until end of life raises concerns about human dignity, the purpose of punishment, and compassionate treatment. There are multiple obstacles to providing effective and compassionate end of life in care in prisons (Maschi et al., 2014), meaning those facing the prospect of remaining in custody to end of life face a ‘double burden’, being both deprived of liberty and having unmet or poorly met health and wellbeing needs (Turner et al., 2018). This is similarly experienced by adults with dementia/MCI, with the prison environment being ill-equipped to handle the complex care needs of people with dementia (Forsyth et al., 2020). Nonetheless, whilst evidence indicates “older adults released from prison have lower recidivism rates than their younger counterparts” (Maschi et al., 2012, p.448), more research is needed to determine how, if at all, cognitive impairment inhibits recidivism, with re-offending risk in an individual with dementia “related to multiple physical, psychiatric and cognitive factors” (Reutens et al., 2024, p.9), and disinhibition known to accompany neurodegenerative dementias (Migliaccio et al., 2020). As a result, solutions such as exempting individuals with dementia from imprisonment are not as viable as they might appear *prima facie*.

Whilst deeper philosophical exploration of these problems is needed, practical approaches must be developed as a priority. To mitigate problems around fitness to plead and enact the Law Commission’s (2016) accepted recommendation to base capacity assessment on current psychiatric understanding and best practice, current Sentencing Council (2020) guidance on *sentencing offenders with mental disorders*,* developmental disorders*,* or neurological impairments* should be strengthened. This guidance might be amended so that courts are legally obliged to consider potential impairment in a formalised, systematic way, with integrated input from medical professionals. The unique effects a prison sentence will have on an individual who may not be always cognisant of self, space, and time should also factor into court decision-making, as should medically informed functional prognostication (Ramsey & Arnold, 2022). Whilst an individual with mild dementia/MCI at the point of sentencing may be able to live a life comparative to their peers in prison, the courts, with medical guidance, should also consider if this will remain the case for the length of the individual’s sentence. Future research into the economic and moral costs of housing older adults in prison until end of life should be prioritised, with a focus on the possible human rights implications (Maschi et al., 2014).

In theory, it is the function of liaison and diversion (L&D) services to ensure individuals engaged in the CJS with mental health difficulties are situated appropriately (Slade et al., 2016). L&D assess charged and/or sentenced individuals, liaising with the appropriate community-based services, and diverting the individual based on assessment of need, if appropriate (Slade et al., 2016). Nonetheless, despite neurocognitive disorders, including types of dementia and MCI, being classified as mental disorders (American Psychiatric Association, 2013; World Health Organisation, 2016), evidence indicates that L&D drastically underserves those with dementia/MCI. An outcome evaluation of the national model for liaison and diversion found dementia, acquired brain injury, and organic mental disorder as needs with very low (< 1%) prevalence in L&D service data (Disley et al., 2021). From two further studies in England and Wales, one reported no data on dementia/MCI referrals from police custody (Forrester et al., 2017), whilst another reported only 0.2% of primary mental health need identified by L&D teams was dementia (McKenna et al., 2019). Additionally, court advocacy provisions for individuals with dementia/MCI also appear to require development (Dixon et al., 2020), with significant confusion arising from differences in relevant legislatures (UK Legislation, 2005, 2007, 2014) which have each introduced their own form of advocacy.

### An unforgiving prison system

Aligned with previous findings (Brooke et al., 2020; Forsyth et al., 2020; Peacock et al., 2020), the rigid, *security-as-priority* ethos which permeates prisons appears to be entirely incompatible with providing the necessary support to individuals with dementia/MCI. Royal College of Nursing (2021) guidance for supporting people with dementia in prison emphasises allowance for an individual’s sensory and mobility needs. Compassion, flexibility, and an environment which is forgiving of a person’s potential confusion or disorientation are also emphasised in research (Brooke et al., 2020; Forsyth et al., 2020; Peacock et al., 2020). However, due to legitimate security concerns, in prison there is no room for compassion when a governor orders lockdown, even if an individual is not aware they are in prison; there is no flexibility in regime times to allow these individuals longer to complete tasks; and adaptations to lessen confusion or disorientation, such as adapted showers or coloured doors, are patchy to non-existent (Forsyth et al., 2020). Despite Public Health England (2016) recommending a more integrated approach to mental illness in prisons, mental health services in English and Welsh prisons are still not considered comprehensive (Forrester et al., 2018). This translates into intense psychological strain for individuals with dementia/MCI, additional to that which is inherent in their condition. Therefore, those with dementia/MCI experience an enhanced burden comparative to their peers.

Facilitators to address this problem have been discussed in other studies (Brooke et al., 2020; Forsyth et al., 2020; Peacock et al., 2020; Treacy et al., 2019), to which the findings of this research generally align. Notably, staff in varied roles spoke of the need for bespoke specialised facilities to accommodate people with dementia/MCI outside of prisons, and considered current ad-hoc facilities to be valuable, if limited in their scope. This is supported by research which reports wide advocacy for the use of compassionate release policies for individuals with severe dementia, or alternative custodial accommodations such as secure nursing homes (Treacy et al., 2024). Nonetheless, despite dementia being named as an example condition in the UK government’s Early Release on Compassionate Grounds Policy Framework (Ministry of Justice and HMPPS, 2023), the minimal evidence available suggests release on compassionate grounds is typically deemed inappropriate for most prisoners, and the stringent prognostic criteria difficult to meet (Turner & Peacock, 2017). Consideration of the impact on victims and their families must also be factored into any compassionate release advocacy for individuals with dementia/MCI. Whilst ethical uncertainty surrounds the detention of individuals who might not remember the reason for their detention, releasing an individual without this recollection and potentially disinhibited behaviours (Migliaccio et al., 2020) also poses a risk to victims and families affected by the offence. Any decisions on compassionate release must balance this carefully.

### An unprepared workforce

The preparedness of staff in prisons to support people with dementia/MCI is currently insufficient. Officers accepted that their limited knowledge in this area prevented them from interacting with individuals with dementia/MCI optimally and lessened their ability to identify signs of dementia/MCI. This sentiment was echoed by healthcare and other support staff. Obvious facilitators to address these systemic training shortfalls include the development and implementation of suitable training and awareness-raising schemes (Perryman et al., 2023). Current training recommendations include prioritising covering the early warning signs of dementia/MCI and increasing awareness of the impact of dementia/MCI on an individual’s ability to function (Cipriani et al., 2017; Perryman et al., 2023); in addition, recently developed bespoke packages for delivering comprehensive training on dementia/MCI in prisons might be adopted (Perryman et al., 2023). This should be combined with awareness-raising efforts for staff and prisoners.

### Lack of collaboration

A lack of collaboration was an apparent barrier to delivering health and social care for individuals with dementia/MCI in prison. As found in other studies (Forsyth et al., 2023; Perryman et al., 2023), the working relationship between various roles in prison, for example between officers and mental health workers, appeared too limited for true collaborative working to be achieved. This situation combines with a lack of understanding of other staff’s roles and responsibilities, and poor communication, to produce an essentially siloed working environment which is incompatible with the multidisciplinary and integrated approach required to identify and support individuals with dementia/MCI (Forsyth et al., 2020; Perryman et al., 2023). Facilitators to connect these siloes should aim to bring together currently separated systems into a more collaborative mode of operation. Changes and improvements in communications systems, understanding of the various teams and their functions, as well as the development of strong working relationships, all hold the potential to pull together various siloes towards improved health and social care provision. To bring about this change, the position of dementia care co-ordinator in prisons has been suggested, whose role would involve connecting the various siloes which have formed (Perryman et al., 2023).

### Peer support ‘plugging the gap’

A notable facilitator to improve health and social care for individuals with dementia/MCI in prison was peer support. These schemes are known to be widely used across prisons to support individuals with a variety of health and social care needs (Stewart, 2011; Walton et al., 2023) and are being adopted across prisons with increasing rapidity (Recoop, 2023). In line with studies identifying the benefit of these schemes for prisons (Bagnall et al., 2015; Stewart & Lovely, 2017), these initiatives were positively appraised by staff and individuals with dementia/MCI. However, as other recent work reported (Cowan et al., 2021; Walton et al., 2023), it was clear that these initiatives differ substantially in formalisation, and that training and regulation are extremely variable. This is especially problematic as the peer support system is potentially open to exploitation by individuals who might not provide adequate support or use their role as a peer supporter for status, gain, or access to vulnerable individuals. Conversely, supporters themselves may be asked to go beyond the duties reasonably expected, be subject to potentially traumatising duties, or be placed in a position to deal with emergencies beyond their trained competency. Solutions to this problem include clear role demarcation, comprehensive training, and regulation (Walton et al., 2023). To establish the value of peer support beyond doubt, empirical evaluation as to the efficacy of these schemes is desperately required (Cowan et al., 2021; Walton et al., 2023).

### Staff ‘hands-tied-behind-back’

Throughout the data, health and social care appeared entirely hamstrung in its approach to dementia/MCI in prisons. Particularly problematic appeared to be the lack of access to specialist facilities, resources, and staff with the expertise to diagnose and manage dementia/MCI. As reported in other studies (Brooke et al., 2020; Forsyth et al., 2020; Peacock et al., 2020), this seemingly translates into a lack of diagnostic, treatment, and support capability for people with dementia/ MCI, leaving staff with their ‘hands tied behind their back’ when dealing with individuals with this condition. Whilst short-term facilitators may take the form of specific solutions to specific problems, for example, access to a CT scanner for brain imagining, or employing a prison-based geriatric psychiatrist; longer-term solutions to this problem likely require a more holistic approach in the form of specialised care units or facilities.

A recent review (Treacy et al., 2024) reported wide endorsement for specialist dementia units across the current literature; however, papers varied significantly in the details of arrangements, with a spectrum of suggestions reported from independent accommodation to 24-hour care including assisted living. One prevalent recommendation across the papers included in the review was for the development of more appropriate accommodation (Treacy et al., 2024); this might be a regional secure facility, potentially oriented towards end-of-life care. Nonetheless, despite the potential benefits of such facilities, there were concerns around “availability, costs and staffing of specialist units, and distances that family would have to travel to visit” (Treacy et al., 2024, p.24). A notable point of debate on specialist facilities is the extent to which such facilities should be integrated with or separate from the general prison population. Several papers have argued that entirely separate wings to support individuals with dementia are more efficacious than integrated units in meeting the health and social care needs of those with dementia (e.g., Du Toit et al., 2019; Maschi et al., 2014; Treacy et al., 2019). However, it was considered important that prisoners and staff were not forced to live and work on these wings, and that opportunities to associate with the general population should be provided (Treacy et al., 2019). Some papers have also suggested benefits to people with dementia/MCI remaining within the general population, including: socialising with younger people; the calming influence of older adults on younger prisoners; keeping people withing their established social networks; and avoiding the potential for the person to feel stigmatised due to their accommodation on a specialist unit (Treacy et al., 2024).

Moreover, whilst there are examples of people with dementia in prison residing in specialist accommodations in England and Wales, these accommodations are not units which are designed specifically to support people with dementia or MCI (Treacy et al., 2024, p.24); rather, they might be designed to accommodate prisoners with disabilities or care needs or be for older adults. Treacy et al. (2024, p.24) report only seven specific units for people with dementia/MCI, and these are all in the United States. At present, no specific dementia/MCI unit or care pathway has been implemented and evaluated in English and Welsh prisons (Forsyth et al., 2020). This kind of research is required to gain a better understanding of what dementia specialist facilities might look like, including operational models, cost-effectiveness, training required, and critically, their impact on people with dementia/MCI in prison. Pathways through the wider CJS might also add value. Solutions which explore L&D services specifically for dementia, dementia-specific arrest processes, and dementia court pathways, might also be developed and similarly evaluated.

### Strengths and limitations

This study gives voice to imprisoned individuals with dementia/MCI across multiple categories of prison, allowing for the consideration of facilitators and barriers to appropriate care in varied settings, how these barriers are felt by the individuals themselves, and the potential personal benefit for these individuals of given facilitators. The study also collates a broad range of perspectives from across a variety of roles throughout and adjacent to the criminal justice system, therefore is well placed to synthesise sophisticated conclusions.

Thematic analysis is widely utilised for its effectiveness in extracting meaningful insights from data. However, inherent features of this method mean findings produced via thematic analysis might vary between researchers. The interpretation of themes is researcher-dependant, with reflexivity and bias potential concerns; and the quality of thematic analysis is skill-dependent, which can also vary between researchers. Accordingly, multiple steps were taken by the research team to establish finding trustworthiness (Nowell et al., 2017). Based on Lincoln and Guba’s evaluative criteria (1985), *credibility* was enhanced by prolonged engagement with the interview transcripts and peer debriefing, including with a senior qualitative researcher; comprehensive description and exposition has been utilised to enable *transferability* to be judged by those who might wish to generalise findings more widely; *dependability* has been enhanced by structured, logical, and traceable analysis facilitated by NVivo software (v.12); and *confirmability* was established by achieving credibility, transferability, and dependability, along with a justifiable methodological approach (Nowell et al., 2017). Researchers were also guided by Braun and Clarke’s (2006, 2012) approach for their analysis and received training in this method.

The study is limited in its geographical scope, with research sites located in northern/midlands England. Nonetheless, this is less of a concern for qualitative research, which does not aim for validity and reliability in the positivist sense quantitative research does. The data of this paper represent rich, important insights which demonstrate what it is like to live with or support those with dementia in criminal justice settings in England and Wales.

Also problematic were the challenges inherent in interviewing individuals with dementia/MCI, such as their ability to comprehend questions and heightened susceptibility to distress and disorientation. Approaches to mitigate this were described in methods (Dewing’s (2007) Process Consent method), though a finding in itself was that multiple interviews with individuals with dementia/MCI were shortened due to their increasing confusion or disorientation casting doubt over their continued consent, or ability to continue the interview without distress.

## Conclusions

Given the rising number of older adults in contact with the CJS (House of Commons Justice Committee, 2020), it is becoming increasingly important to consider the way in which individuals with dementia/MCI are supported. Ethical concerns around the judicial process for individuals with diminished cognitive capacity are complex, nonetheless must be considered and addressed; likewise, those who run prisons must consider ways to make the living environment more appropriate for these individuals. To accomplish this, staff must be appropriately trained to support and identify individuals with dementia/MCI in prison, and awareness of dementia/MCI must be raised throughout these establishments. The presently siloed ways of working must also be addressed, so that a joined-up collaborative approach to health and social care for these individuals is adopted. Specialised wings or units designed to support individuals with higher health and social care needs, such as those with dementia/MCI, may provide a more holistic solution, but more evidence is needed towards their development. In the interim, well-defined multidisciplinary dementia care pathways should be developed and adopted as a priority. Lastly, the peer support schemes which currently buttress health and social care arrangements in prisons require formal evaluation, and training/oversight of these schemes should be comprehensive and standardised.

## Data Availability

The datasets generated and/or analysed during the current study are not publicly available for reasons including potential re-identifiability of qualitative responses to interview questions. Consent for data sharing was not granted by study participants.
